# BioAssemblyModeler (BAM): User-Friendly Homology Modeling of Protein Homo- and Heterooligomers

**DOI:** 10.1371/journal.pone.0098309

**Published:** 2014-06-12

**Authors:** Maxim V. Shapovalov, Qiang Wang, Qifang Xu, Mark Andrake, Roland L. Dunbrack

**Affiliations:** Institute for Cancer Research, Fox Chase Cancer Center, Philadelphia, Pennsylvania, United States of America; University of Alberta, Canada

## Abstract

Many if not most proteins function in oligomeric assemblies of one or more protein sequences. The Protein Data Bank provides coordinates for biological assemblies for each entry, at least 60% of which are dimers or larger assemblies. BioAssemblyModeler (BAM) is a graphical user interface to the basic steps in homology modeling of protein homooligomers and heterooligomers from the biological assemblies provided in the PDB. BAM takes as input up to six different protein sequences and begins by assigning Pfam domains to the target sequences. The program utilizes a complete assignment of Pfam domains to sequences in the PDB, PDBfam (http://dunbrack2.fccc.edu/protcid/pdbfam), to obtain templates that contain any or all of the domains assigned to the target sequence(s). The contents of the biological assemblies of potential templates are provided, and alignments of the target sequences to the templates are produced with a profile-profile alignment algorithm. BAM provides for visual examination and mouse-editing of the alignments supported by target and template secondary structure information and a 3D viewer of the template biological assembly. Side-chain coordinates for a model of the biological assembly are built with the program SCWRL4. A built-in protocol navigation system guides the user through all stages of homology modeling from input sequences to a three-dimensional model of the target complex. Availability: http://dunbrack.fccc.edu/BAM.

## Introduction

High-throughput methods in proteomics have resulted in substantial information on protein-protein interactions and identification of the components of large protein complexes [1]. The structures of these interactions are important for understanding mechanisms in various pathways and the effects of mutations observed in human populations. In addition, many proteins are oligomeric due to the association of identical subunits under physiological conditions [2].

As with predicting the structure of a single protein, homology modeling of complexes, when suitable templates exist, is very likely to produce better models than ab initio prediction via computational docking [3]. Template-based structure prediction of binary complexes has been shown to predict accurate models for 65% of cases with easily-identified homologous templates.[4] Kundrotas et al. have shown that when structures of the monomers (or easily-identified homologues thereof) are available, templates for the complex exist in a large majority of cases.[5] It is therefore valuable to mine as much data on protein interactions as possible from experimental structures, and to use these structures as templates for modeling particular target complexes of interest. In recent years, the number of structures in the PDB has grown rapidly, and the structures have increased in complexity and diversity, providing a valuable resource for modeling of protein interactions.

For X-ray structures, the PDB provides Cartesian coordinates for asymmetric units and for biological assemblies. The asymmetric unit is defined as the smallest portion of a crystal structure that can be used to model the entire crystal using crystallographic symmetry operators. This is in contrast to the biological assembly, which represents a hypothetical biologically active structure which may be present within the crystal. These biological assemblies may be identical with the asymmetric unit (∼50% of PDB entries [6]), substructures of the asymmetric unit (∼25%), or larger than the asymmetric unit (∼25%) built by applying symmetry operations from the crystallographic space group. The PDB's biological assemblies are those deposited by the authors of new structures or as determined by the program PISA [7]. Although biological assemblies from the authors and PISA are often hypothetical, these data are potential sources of information for modeling the biological assemblies of proteins.

We have developed a downloadable program, BioAssemblyModeler or BAM that provides for the modeling of the structures of protein homo- and heterooligomers. While some webservers allow for the modeling of hetero- and homodimers [8]–[10], BAM models protein complexes of up to six sequences and includes multiple subunits of each sequence according to their arrangement in the template biological assembly. BAM assigns Pfam domains to the queries and finds templates and the content of their biological assemblies from our regularly updated PDBfam database [11]. It uses profile-profile alignment of the targets and the templates [12] and SCWRL4 [13] for modeling the coordinates of the protein complexes, all in a visual environment. The interface of BAM is user-friendly, intuitive and streamlined. We included a beginner mode which navigates a user through a typical homology modeling protocol showing what to do next by highlighting relevant controls: menu items, buttons, textboxes and messages in the status bar and popup boxes. Within 10–60 minutes the user can expect to produce a homology model of a biologically active protein complex saved in PDB format.

## Methods

BAM is a Windows application that runs on both the 32-bit or 64-bit editions of Windows XP, Vista, Windows 7 and Windows 8. It was written in C# with Microsoft Visual Studio. Windows can be installed on Linux and all recent Macintosh machines using free virtualization software such as VirtualBox (http://virtualbox.org). We have tested BAM using VirtualBox (http://www.virtualbox.org) on both Linux and Mac OS and it functions well. The BAM graphical installer [49 MB] is available for free download. With a few mouse clicks the installer automatically extracts and configures initial databases, utilities, and software required. In Beginner mode, BAM informs a user about the required actions at each step by means of message boxes, highlighting of different user controls in BAM's graphical user interface (GUI) and giving instructions in a status bar at the window bottom. The beginner mode can be disabled in Tools->Settings. Advanced settings of BAM and third-party dependencies can be adjusted in Tools-> Settings->View or Edit.

BAM relies on two data files: a relational database (PDBfam) and a large set of protein sequences formatted for use by PSI-BLAST (such as Uniref [14], NCBI's nr [15], or pdbaa [16], [17]). The relational database from our web site includes Pfam assignments to nearly all proteins in the PDB and information on the contents of biological assemblies for each entry. The sequence file is used to build PSI-BLAST profiles for each target sequence. By default a smaller database, pdbaa containing only PDB sequences is already included with the BAM distribution. This file is adequate for target sequence closely related to proteins in the PDB. During the first launch BAM suggests downloading PDBfam database [250 MB] and at user's discretion a larger sequence database such as Uniref50 [1.8 GB] or Uniref90 [4.0 GB]. The larger sequence database leads to more accurate results for targets that are only remotely related to PDB structures. However, it takes more computational time during alignment steps. It is also recommended to update these databases every few months, via Main Menu->Tools->Download/Update Databases.

In addition the user can update/reinstall BAM itself at any time (Tools -> Reinstall/Update BAM). Whenever a new version of BAM or PDBfam database update are available, the user will be automatically notified through the BAM user interface. In addition BAM relies on a set of online third-party services like RCSB PDB or Sanger Pfam. Problems may occur once these providers make changes to their formats or service. The BAM updates will comply with any future format changes in the PDB or Pfam that may arise.

Homology modeling of biological assemblies with BAM can be accomplished with the following eight steps. The user is advised to refer to video and screenshot tutorials and more detailed information on how to use BAM available at http://dunbrack.fccc.edu/BAM.

(1) To start a new homology modeling project, a user needs to open for editing a FASTA-formatted sequence file with one or more different amino acid sequences of a target protein complex. Even when a homooligomer model is desired, only one copy of the sequence is required in the input file. Sample sequence files are included in the BAM installer for learning and testing purposes. BAM automatically checks the user's FASTA sequence input for any inconsistencies and advises if any are detected.

(2) PSI-BLAST (Altschul, et al., 1997) is run with two or more rounds to generate a sequence profile for each target sequence - a matrix of 20 amino acid types by the sequence length, giving a log odds score of finding a particular amino acid in each position of a multiple sequence alignment of the target sequence family. These profile matrices are required for secondary structure (SS) prediction and profile-profile alignment of the target and template sequences.

(3) PsiPred (Jones, 1999) is run next to predict the secondary structure of each target sequence for each PSI-BLAST run. The results can be viewed within BAM – red for helix, green for sheet, and gray for coil. Higher color intensity corresponds to higher confidence of prediction.

(4) Each target sequence is submitted to the Pfam web service [18] to find and assign family domains with functional annotation. The number, order, locations, and names of these Pfam domains on each target sequence constitute the target sequence domain architecture. Any assigned domain can be clicked to open Pfam domain Wikipedia page.

(5) A search for the target Pfams is performed on a precompiled relational database, based on PDBfam [11], which contains assigned Pfam domains for all PDB entries. The number of PDB structures belonging to each template architecture is shown next to it. The list is ordered based on the similarity of the template architectures and the target architecture. If not all target domains are found, the most similar architectures will still be shown.

(6) A user selects one or more template architectures and presses a Compute button to perform profile-profile sequence alignments [12] of each target and template protein sharing at least one Pfam domain. In the background, BAM downloads precomputed profiles for the PDB sequences from our server. The hit table has a row for each potential template biological assembly and a set of columns for the assembly (resolution, sequence assembly architecture and chain assembly architecture) and for each target sequence (sequence alignment identity, alignment gap percentage, alignment length, alignment start/end positions). If more than one target sequence shares the same Pfam, BAM assigns the target sequence to the template sequence with the highest sequence identity. The list is first sorted by target-template Pfam architecture similarity and then by sequence identity of each target-template sequence pair. The list can be resorted by clicking any column and the default sorting can also be restored.

(7) Double-clicking any row from the template hit table opens a form with the content of the biological assembly with assigned Pfam domains of each chain copy. After confirming the choice, BAM automatically downloads a template structure in XML format from the PDB, applies symmetry operators in the file to build a biological assembly, and opens a visualization window that contains a rotatable view of the structure. The alignments depict the predicted secondary structure of the targets and the experimental secondary structure of the template chains. The start/end positions, deletions and insertions are shown in the viewer, and are dynamically updated as the alignment is edited. The user can choose which chains to include into a target complex model so that substructures of the assembly can be modeled. The user is advised to try several templates and review biological assembly content, alignment identities, and locations of gaps until a satisfactory template is found.

(8) The model is built by clicking on a button that copies the template backbone to a new PDB file with the sequence numbered according to the edited alignments, and clicking another button to run our side-chain prediction program SCWRL4 [13], which models all of the mutated side chains with a backbone-dependent rotamer library [19], preserving the rotamers of residues that are identical in the target and template sequences. The last action produces a model of the target complex in PDB format. BAM does not perform loop modeling or model refinement, which can be carried out with other programs using the BAM model as input.

## Results and Discussion

**Figure 1:**
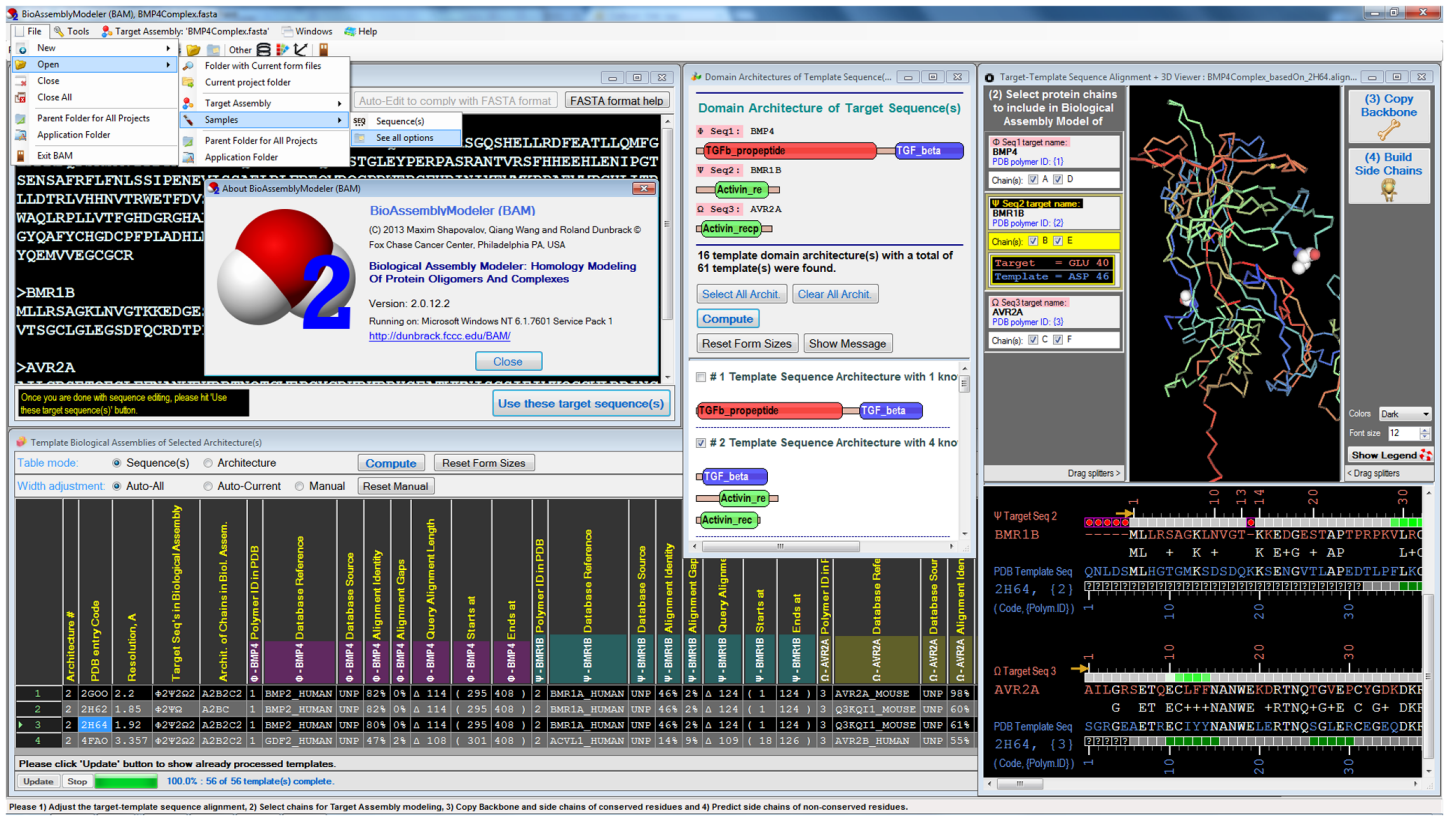
Screenshot of BAM showing a modeling session of a heterohexameric complex. For details, please refer to Results and Discussion section.

An example session of modeling a heterohexameric complex is shown in Figure 1. The target sequences (upper left) consist of the human BMP4 and the extracellular domains of its potential type 1 and type 2 receptors BMR1B and AVR2A respectively [20]. The middle window at top (“Domain Architectures of Template Sequences”) shows that the target sequence BMP4_HUMAN contains Pfam domains (TGFb_propeptide) and (TGF_beta) while the receptors both consist of (Activin_rec) domains. The first template architecture contains both domains of the BMP4 protein while the second architecture contains one of its domains but with two other proteins with (Activin_rec) domains. Filling the template table at lower left with PDB entries from this architecture shows a number of biological assemblies that can be built. The PDB entry 2H64 contains the proper TGF_beta domain dimer and two copies of each of the two receptor domains, producing a heterohexameric assembly. From the profile-profile alignments, BAM determines that BMR1B is more closely related (46% identity) to entity sequence 2 (BMR1A_HUMAN) in 2H64 than it is to entity sequence 3 (Q3KQI1_MOUSE, which is identical in sequence with AVR2B_HUMAN), while AVR2A is closer to entity sequence 3 than entity sequence 2. The sequence alignments (lower right) are shown with a rotatable view of the template complex, and can be edited by dragging gap characters on the alignment. The locations of insertions and deletions are marked on the structure and are dynamically updated as the sequence alignments are edited. Chains to be included in the model can be removed by unchecking the check boxes (upper left of the “Target-Template Sequence Alignment” window at right). A model is produced by clicking the “Copy backbone” and “Build side chains” buttons on the upper right of the window at right.

BAM presents a straightforward modeling procedure for assemblies containing up to six different sequences. These assemblies can be oligomers of any size, as dictated by the biological assemblies recorded in the XML files of each PDB entry. Such assemblies should be used with caution since they do not always correspond to what authors describe in papers and in some cases authors may fail to recognize the presence of the correct oligomeric assembly within their crystals [21]. In other cases, the correct oligomer may not be known and may be difficult to recognize. Our ProtCID database, which contains common interfaces across crystal forms of homologous proteins and protein pairs, may provide further information on which structures contain the correct biological assembly [22].

One advantage of BAM is that all available templates are presented, and a number of models can be produced and compared. The alternatives are laid out in the template hit table. Another strong side of BAM is that it presents its templates in terms of their biological contents in the form of Pfam domains. Many modeling web servers and programs simply present a list of templates with their PDB codes and perhaps the protein names but the user has no way of knowing whether the hits all come from the same protein family and whether they would produce the same protein folds or entirely different ones. Because BAM finds the Pfams of the target and the Pfams of the PDB proteins in separate steps, the procedure is effectively an intermediate sequence profile search [23], where the intermediate profile is the Pfam HMM. This is an effective way of finding remote homologues in a computationally efficient manner. It will not find all remote homologues for the targets, in cases where the targets or templates are in different Pfams within the same Pfam clan and in a small number of cases for which Pfam lacks a domain definition for a protein in the PDB. This is relatively rare, and the advantages of organizing the template biological assemblies by domain content seem to outweigh these rare situations in our view. Future versions of BAM will include a direct search of the PDB to handle such cases.

**Figure 2:**
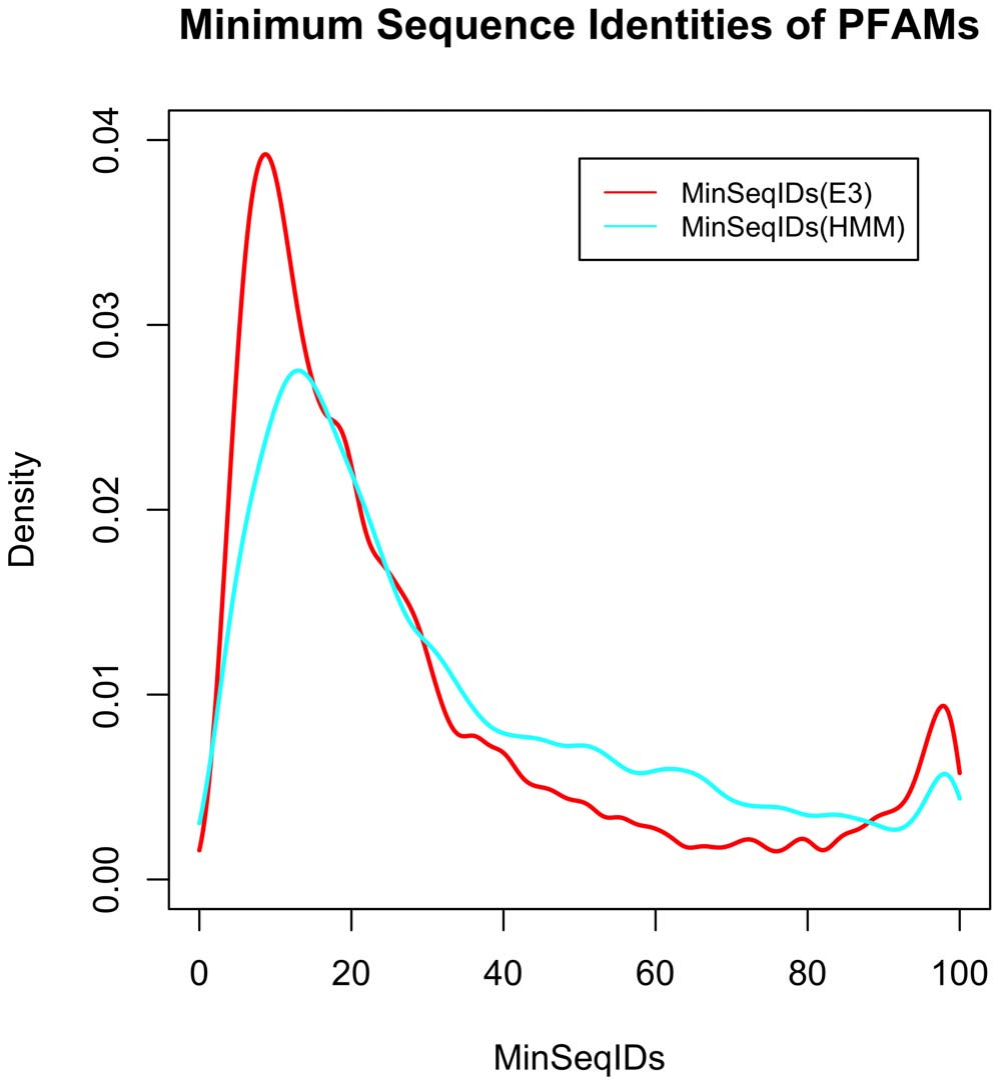
Minimum sequence identity within Pfam domain families identified in PDB structures with PDBfam. Kernel density estimates of the minimum sequence identifies are shown for a total of 3730 Pfams. The sequence identities were determined by alignment of pairs of PDB sequences to the same HMM (curve labeled HMM) or by structure alignment with the program FATCAT.

To get an idea of the relationships that can be identified via HMM-sequence alignments of the target and template sequences to the same Pfam HMM, we produced a density estimate of the sequence identities of PDB sequence pairs in the same Pfam as identified in our PDBfam database [11]. PDBfam uses both structure alignments with FATCAT [24] and alignments of PDB sequences to the Pfam HMMs. It currently assigns 6,901 different Pfams to at least one sequence in the PDB and 15,011 pairs of Pfams (both intrachain and interchain) present in at least one entry in the PDB. The sequence identities of the HMM alignments can be determined by counting identical and non-identical residues aligned to the same HMM positions for each of two PDB sequences (i.e., a transitive alignment). The results are shown in Figure 2, which plots the minimum sequence identity for each of 3730 Pfams as determined by FATCAT with an E-value cutoff of 1.0e-3 and by the HMM transitive alignments. The FATCAT alignments are skewed to lower sequence identities than the HMM alignments, because they align greater proportions of the structures than the local alignments provided by the HMMs. We found this to be the case in developing our PISCES server, for which structure alignments are often longer and lower sequence identity than PSI-BLAST alignments for the same pair of sequences [17]. Nevertheless, the HMM alignments have a peak at 16%, showing that relatively distant homology relationships can be identified by alignment to the same Pfam HMMs. The accuracy of our profile-profile alignment algorithm has been previously benchmarked [12].

Another important aspect of modeling oligomers is whether the target sequences are likely to share the same interfaces and biological assemblies as the templates. This is a complicated issue, because we have previously shown that the annotated biological assemblies in the PDB are only 80–90% accurate [21]. One advantage of BAM is that it shows the user all of the available templates and allows for rapid modeling of oligomeric structures from many different templates, so that even if the biological assembly for one PDB entry is incorrect (usually because it is smaller than the correct oligomer) this will become evident of models are made from multiple templates.

We developed our ProtCID database to investigate just this issue [22]. When the same interface is present in multiple crystal forms of the same protein or members of the same family (or families for heterodimers), then the interface is likely to be part of the correct biological assembly. This is especially true when the sequence identity of proteins in the same cluster is below 90%. In our benchmarking study, we found that when there are at least 5 different crystal forms and the minimum sequence identity is below 90%, there is a strong agreement that the interfaces present in the cluster are parts of the biological assembly [21].

**Figure 3:**
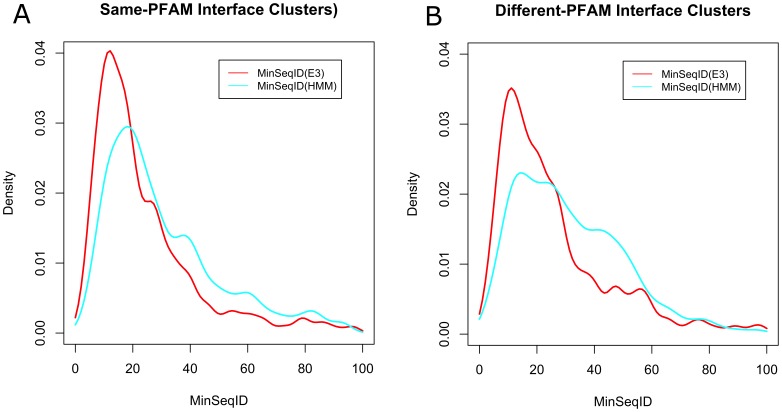
Minimum sequence identities in ProtCID clusters of common interfaces between identical Pfams (A) or different Pfams (B). The minimum sequence identity for each pair of domains in the same interface cluster using both FATCAT and transitive alignment via the Pfam HMM(s). For Pfam pairs, the minimum of the two Pfams was used in the density estimate. Same-Pfam clusters may contain homodimers and/or heterodimers that belong to the same Pfam domain family.

To examine this for the current PDB, we calculated the minimum sequence identity of Pfam-Pfam domain interactions in ProtCID clusters with at least 5 crystal forms and minimum sequence identity below 90%, as determined by sequence alignment or structure alignment. The results for same Pfam-Pfam interface clusters and heterodimeric Pfam-Pfam interface clusters are shown in Figure 3A and 3B respectively. Again, the sequence identities obtained from FATCAT structure alignments and HMM alignments are shown. The peak in the densities for the HMM alignments are at about 20% and 18% for same- and different-Pfam clusters. Again, FATCAT is able to determine lower sequence identities of about ∼12% because of longer alignments. The results indicate that biological interactions can be preserved in homologous proteins down to very low sequence identities.

Finally, we used ProtCID to identify well-documented cases where Pfam domain-domain interactions may in fact be different for homologous proteins or pairs of proteins. We found 1801 Pfams or pairs of Pfams for which there is at least one interface clusters comprising 5 or more crystal forms with minimum sequence identity <90%. Of these, 572 clusters (32%) had two or more such clusters and only 115 (6%) had no overlap of PDB entries or crystal forms between the two largest clusters. The remaining 457 had entries in both clusters and are presumably components of oligomers larger than dimers. It is an important point that some domains may interact in more than one way but may do so only as part of the same oligomer, rather than reflecting mutually exclusive interactions. When biological assemblies in the PDB are incorrect, it may appear as if two homologous proteins have evolved different dimer interactions, when in fact both crystals may contain the same larger oligomer. Not accounting for this may lead to some underestimation of the extent of interface conservation [25].

Despite these caveats, the utility of modeling biological assemblies has been made evident in our experience in collaborating with colleagues in applying molecular modeling to real-world biological problems [26]–[29], and we hope that users will also find the new program BAM helpful.

## References

[1] Koh GC, Porras P, Aranda B, Hermjakob H, Orchard SE (2012) Analyzing protein-protein interaction networks. J Proteome Res 11: 2014–2031. 22385417 10.1021/pr201211w

[2] Traut TW (1994) Dissociation of enzyme oligomers: a mechanism for allosteric regulation. Crit Rev Biochem Mol Biol 29: 125–163. 8026214 10.3109/10409239409086799

[3] Gray JJ, Moughon S, Wang C, Schueler-Furman O, Kuhlman B, et al (2003) Protein-protein docking with simultaneous optimization of rigid-body displacement and side-chain conformations. J Mol Biol 331: 281–299. 12875852 10.1016/s0022-2836(03)00670-3

[4] Mosca R, Ceol A, Aloy P (2013) Interactome3D: adding structural details to protein networks. Nat Methods 10: 47–53. 23399932 10.1038/nmeth.2289

[5] Kundrotas PJ, Zhu Z, Janin J, Vakser IA (2012) Templates are available to model nearly all complexes of structurally characterized proteins. Proc Natl Acad Sci U S A 109: 9438–9441. 22645367 10.1073/pnas.1200678109 PMC3386081

[6] Xu Q, Canutescu A, Obradovic Z, Dunbrack RL Jr (2006) ProtBuD: a database of biological unit structures of protein families and superfamilies. Bioinformatics 22: 2876–2882. 17018535 10.1093/bioinformatics/btl490

[7] Krissinel E, Henrick K (2007) Inference of macromolecular assemblies from crystalline state. J Mol Biol 372: 774–797. 17681537 10.1016/j.jmb.2007.05.022

[8] Fukuhara N, Kawabata T (2008) HOMCOS: a server to predict interacting protein pairs and interacting sites by homology modeling of complex structures. Nucleic Acids Res 36: W185–189. 18442990 10.1093/nar/gkn218 PMC2447736

[9] Kittichotirat W, Guerquin M, Bumgarner RE, Samudrala R (2009) Protinfo PPC: a web server for atomic level prediction of protein complexes. Nucleic Acids Res 37: W519–525. 19420059 10.1093/nar/gkp306 PMC2703994

[10] Mukherjee S, Zhang Y (2011) Protein-protein complex structure predictions by multimeric threading and template recombination. Structure 19: 955–966. 21742262 10.1016/j.str.2011.04.006 PMC3134792

[11] Xu Q, Dunbrack RL Jr (2012) Assignment of protein sequences to existing domain and family classification systems: Pfam and the PDB. Bioinformatics 28: 2763–2772. 22942020 10.1093/bioinformatics/bts533 PMC3476341

[12] Wang G, Dunbrack RL Jr (2004) Scoring profile-to-profile sequence alignments. Protein Sci 13: 1612–1626. 15152092 10.1110/ps.03601504 PMC2279992

[13] Krivov GG, Shapovalov MV, Dunbrack RL Jr (2009) Improved prediction of protein side-chain conformations with SCWRL4. Proteins 77: 778–795. 19603484 10.1002/prot.22488 PMC2885146

[14] Magrane M, Consortium U (2011) UniProt Knowledgebase: a hub of integrated protein data. Database (Oxford) 2011: bar009. 21447597 10.1093/database/bar009 PMC3070428

[15] Sayers EW, Barrett T, Benson DA, Bolton E, Bryant SH, et al (2012) Database resources of the National Center for Biotechnology Information. Nucleic Acids Res 40: D13–25. 22140104 10.1093/nar/gkr1184 PMC3245031

[16] Wang G, Dunbrack RL Jr (2003) PISCES: a protein sequence culling server. Bioinformatics 19: 1589–1591. 12912846 10.1093/bioinformatics/btg224

[17] Wang G, Dunbrack RL Jr (2005) PISCES: recent improvements to a PDB sequence culling server. Nucleic Acids Res 33: W94–98. 15980589 10.1093/nar/gki402 PMC1160163

[18] Punta M, Coggill PC, Eberhardt RY, Mistry J, Tate J, et al (2012) The Pfam protein families database. Nucleic Acids Res 40: D290–D301. 22127870 10.1093/nar/gkr1065 PMC3245129

[19] Shapovalov MV, Dunbrack RL Jr (2011) A smoothed backbone-dependent rotamer library for proteins derived from adaptive kernel density estimates and regressions. Structure 19: 844–858. 21645855 10.1016/j.str.2011.03.019 PMC3118414

[20] Nickel J, Sebald W, Groppe JC, Mueller TD (2009) Intricacies of BMP receptor assembly. Cytokine & growth factor reviews 20: 367–377. 19926516 10.1016/j.cytogfr.2009.10.022

[21] Xu Q, Canutescu AA, Wang G, Shapovalov M, Obradovic Z, et al (2008) Statistical analysis of interface similarity in crystals of homologous proteins. J Mol Biol 381: 487–507. 18599072 10.1016/j.jmb.2008.06.002 PMC2573399

[22] Xu Q, Dunbrack RL Jr (2011) The protein common interface database (ProtCID) –a comprehensive database of interactions of homologous proteins in multiple crystal forms. Nucleic Acids Res 39: D761–770. 21036862 10.1093/nar/gkq1059 PMC3013667

[23] Soding J, Remmert M (2011) Protein sequence comparison and fold recognition: progress and good-practice benchmarking. Curr Opin Struct Biol 21: 404–411. 21458982 10.1016/j.sbi.2011.03.005

[24] Ye Y, Godzik A (2003) Flexible structure alignment by chaining aligned fragment pairs allowing twists. Bioinformatics 19 Suppl 2II246–II255. 14534198 10.1093/bioinformatics/btg1086

[25] Hamp T, Rost B (2012) Alternative protein-protein interfaces are frequent exceptions. PLoS computational biology 8. 10.1371/journal.pcbi.1002623 PMC3410849 22876170

[26] Li C, Andrake M, Dunbrack R, Enders GH (2010) A bifunctional regulatory element in human somatic Wee1 mediates cyclin A/Cdk2 binding and Crm1-dependent nuclear export. Mol Cell Biol 30: 116–130. 19858290 10.1128/MCB.01876-08 PMC2798281

[27] Wei Q, Wang L, Wang Q, Kruger WD, Dunbrack RL Jr (2010) Testing computational prediction of missense mutation phenotypes: functional characterization of 204 mutations of human cystathionine beta synthase. Proteins 78: 2058–2074. 20455263 10.1002/prot.22722 PMC3040297

[28] Roberts JL, Buckley RH, Luo B, Pei J, Lapidus A, et al (2012) CD45-deficient severe combined immunodeficiency caused by uniparental disomy. Proceedings of the National Academy of Sciences 109: 10456–10461. 10.1073/pnas.1202249109 PMC3387083 22689986

[29] Wei Q, Xu Q, Dunbrack RL (2013) Prediction of phenotypes of missense mutations in human proteins from biological assemblies. Proteins: Structure, Function, and Bioinformatics 81: 199–213. 10.1002/prot.24176 PMC3552143 22965855

